# Omics and Computational Modeling Approaches for the Effective Treatment of Drug-Resistant Cancer Cells

**DOI:** 10.3389/fgene.2021.742902

**Published:** 2021-10-06

**Authors:** Hae Deok Jung, Yoo Jin Sung, Hyun Uk Kim

**Affiliations:** ^1^ Department of Chemical and Biomolecular Engineering (BK21 four), Korea Advanced Institute of Science and Technology (KAIST), Daejeon, South Korea; ^2^ KAIST Institute for Artificial Intelligence, KAIST, Daejeon, South Korea; ^3^ BioProcess Engineering Research Center and BioInformatics Research Center KAIST, Daejeon, South Korea

**Keywords:** cancer, drug resistance, omics, computational modeling, network-based model, machine learning, genome-scale metabolic model

## Abstract

Chemotherapy is a mainstream cancer treatment, but has a constant challenge of drug resistance, which consequently leads to poor prognosis in cancer treatment. For better understanding and effective treatment of drug-resistant cancer cells, omics approaches have been widely conducted in various forms. A notable use of omics data beyond routine data mining is to use them for computational modeling that allows generating useful predictions, such as drug responses and prognostic biomarkers. In particular, an increasing volume of omics data has facilitated the development of machine learning models. In this mini review, we highlight recent studies on the use of multi-omics data for studying drug-resistant cancer cells. We put a particular focus on studies that use computational models to characterize drug-resistant cancer cells, and to predict biomarkers and/or drug responses. Computational models covered in this mini review include network-based models, machine learning models and genome-scale metabolic models. We also provide perspectives on future research opportunities for combating drug-resistant cancer cells.

## Introduction

Drug resistance has been a major obstacle for a successful treatment of cancers, as manifested by over 90% mortality of cancer patients that appeared to be associated with drug resistance (Bukowski et al., 2020). Drug resistance is a phenotypic state that arises as a result of a complex interplay between genetic and non-genetic mechanisms (Marine et al., 2020). Such genetic and non-genetic reprogramming consequently leads to drug resistance through various mechanisms (Gatti and Zunino, 2005; Housman et al., 2014; Zheng, 2017; Lim and Ma, 2019; Vasan et al., 2019; Bukowski et al., 2020), including: drug inactivation, for example by an excessive level of glutathione that detoxifies xenobiotics (Jiang et al., 2017; De Luca et al., 2019); alteration of a drug target by mutations or changes in an expression level (Likhite et al., 2006; Costa et al., 2008); drug efflux by transporters (Giddings et al., 2021); enhanced DNA damage repair system (Harte et al., 2014); development of resistance via dysregulated autophagy (Martin et al., 2017; Cai et al., 2019); epithelial-mesenchymal transition (EMT) (Fischer et al., 2015; Zheng et al., 2015); or heterogeneity of a cancer cell population having cancer stem cells (Seth et al., 2019; Zhao et al., 2021). A state of drug resistance is indeed a highly complex phenotype that requires multidimensional approaches.

Omics technologies have now become indispensable for characterizing mechanisms of cancer progression, and for identifying effective biomarkers and treatment targets for cancers. For this reason, large-scale projects have been launched to generate omics data of various cancer cells. A recent representative example is the Pan-Cancer Analysis of Whole Genomes (PCAWG) Consortium of the International Cancer Genome Consortium (ICGC) and The Cancer Genome Atlas (TCGA), which has allowed advanced studies on gene mutations and gene expression profiles across cancers (Consortium, 2020). The resulting various datasets from such large-scale efforts have been found to be useful for studying drug-resistant cancer cells. Relevant representative datasets include the NCI-60 Human Tumor Cell Lines Screen (Shoemaker, 2006), the Genomics of Drug Sensitivity in Cancer (GDSC) (Yang et al., 2013), TCGA (Cancer Genome Atlas Research et al., 2013), the Cancer Therapeutic Response Portal (CTRP) (Seashore-Ludlow et al., 2015), L1000 profiles from The Library of Integrated Network-Based Cellular Signatures (LINCS) Program (Subramanian et al., 2017), the Cancer Cell Line Encyclopedia (CCLE) (Ghandi et al., 2019), and the Catalogue Of Somatic Mutations In Cancer (COSMIC) (Tate et al., 2019). All these datasets have served as a source of novel insights that help characterize and overcome drug-resistant cancer cells. In particular, it is expected that an increasing volume of such large-scale datasets will facilitate development of various computational models that will better systematize our approaches to studying drug-resistant cancer cells.

**FIGURE 1:**
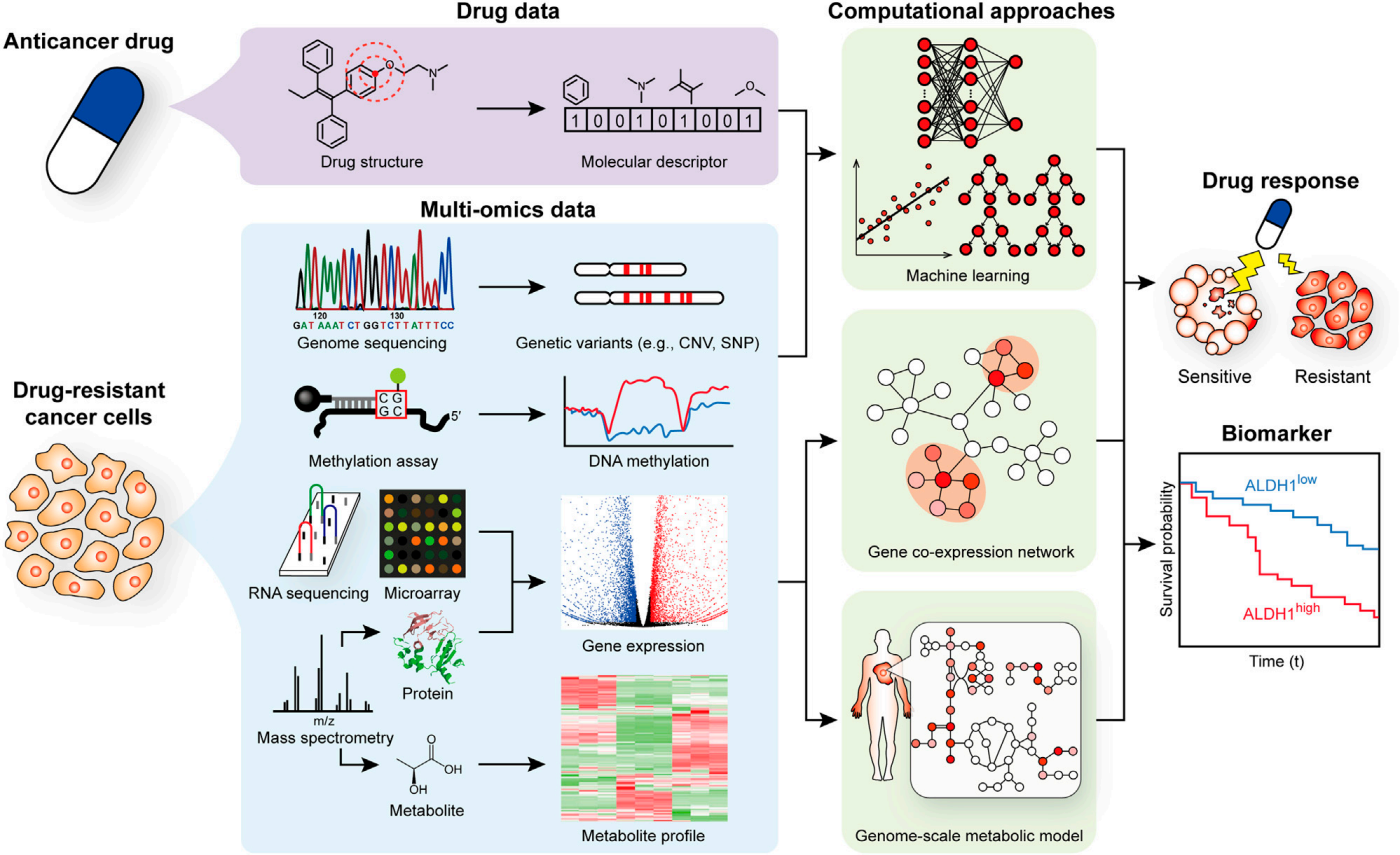
Scheme of omics data generation and computational modeling to better understand and treat drug-resistant cancer cells.

We here review recent studies that utilized multi-omics and computational modeling approaches to better understand mechanisms associated with the progression of drug resistance, and to identify biomarkers and/or drug responses (Figure 1 and Table 1). Especially, we put more focus on computational modeling that makes predictions for various scenarios for the treatment of drug-resistant cancer cells. We also provide an outlook for further advances on the use of computational models for studying drug-resistant cancer cells.

**TABLE 1:** Recent studies on the use of omics data and computational models to better understand and treat drug-resistant cancer cells.

Approaches	Cancer types	Resistance type	Objectives	Drugs	References
Multi-omics analyses					
• ChiP-seq	• Lung cancer	• Both acquired and intrinsic resistance	• Identification of biomarkers	• Erlotinib, osimertinib, crizotinib, vemurafenib, celastrol, and GSK-1059615	Aissa et al. (2021)
• Single-cell RNA-seq					
• RNA-seq					
• Proteome (LC-MS/MS)					
• ATAC-seq	• Breast cancer	• Intrinsic resistance	• Biological characterization	• Doxorubicin	Kumar et al. (2021)
• RNA-seq			• Identification of therapeutic targets		
• Genome sequencing	• 101 Types of cancers from 40,848 patients from cBioPortal	• Not specified	• Biological characterization	• MAPK pathway inhibitors (e.g., selumetinib)	Sinkala et al. (2021)
• Methylome (reduced representation bisulfite sequencing)					
• mRNA microarray and RNA-seq					
• RNA-seq	• Melanoma	• Intrinsic resistance	• Biological characterization	• Vemurafenib	Torre et al. (2021)
• Pooled CRISPR screen (MiSeq)					
• Genome sequencing	• Breast cancer	• Acquired resistance	• Biological characterization	• Tamoxifen and fulvestrant	Achinger-Kawecka et al. (2020)
• Methylome (bisulfite sequencing)					
• Hi-C					
• ChiP-seq					
• RNA-seq					
• Methylome (EPIC array)	• Breast cancer	• Acquired resistance	• Biological characterization	• Paclitaxel	Deblois et al. (2020)
• ChiP-seq					
• RNA-seq					
• Metabolome (LC-HRMS)					
• ATAC-seq	• Head and neck squamous carcinoma	• Acquired resistance	• Biological characterization	• Cetuximab	Kagohara et al. (2020)
• Single-cell RNA-seq					
• RNA-seq					
• Translatome (microarray)	• Leukemia	• Not specified	• Biological characterization	• Cytosine arabinoside	Lee et al. (2020)
• mRNA microarray			• Identification of therapeutic targets		
• Proteome (LC-MS/MS)					
• Genome sequencing	• Breast cancer	• Acquired resistance	• Biological characterization	• Navitoclax	Marczyk et al. (2020)
• Methylome (bisulfite sequencing)			• Identification of biomarkers		
• ATAC-seq					
• Single-cell RNA-seq					
• RNA-seq					
• ChiP-seq	• Breast cancer	• Acquired resistance	• Biological characterization	• Doxorubicin and 5-fluorouracil (5-FU)	Mukherjee et al. (2020)
• RNA-seq					
• Single-cell RNA-seq	• Lung cancer	• Acquired resistance	• Biological characterization	• Cisplatin and paclitaxel	Poojan et al. (2020)
• RNA-seq	• Gastric cancer				
• ATAC-seq	• Leukemia	• Acquired resistance	• Biological characterization	• Bromodomain and Extra-Terminal motif (BET) inhibitor	Bell et al. (2019)
• ChiP-seq					
• Single cell RNA-seq					
			• Identification of therapeutic targets		
• RNA-seq					
• Click-seq					
• RNA-seq	• Lymphoma	• Not specified	• Identification of biomarkers	• Anthracycline-based regimen R-CHOP (i.e., rituximab, cyclophosphamide, doxorubicin, vincristine and prednisone)	Fornecker et al. (2019)
• Proteome (nanoLC-MS/MS)					
			• Identification of therapeutic targets		
• Proteome, phosphoproteome, kinome (LC-MS/MS)	• Breast cancer	• Acquired resistance	• Biological characterization	• 2,5-​diaziridinyl-3-hydroxyl-6-methyl-1,4-benzoquinone (RH1)	Kuciauskas et al. (2019)
• ChiP-seq	• Breast cancer	• Intrinsic resistance	• Biological characterization	• Trastuzumab	Nava et al. (2019)
• RNA-seq			• Identification of biomarkers		
• Exome sequencing	• Breast cancer	• Both acquired and intrinsic resistance	• Biological characterization	• Epirubicin, docetaxel, and bevacizumab	Kim et al. (2018)
• Single-cell DNA-seq					
• Single-cell RNA-seq					
• Genome sequencing• mRNA microarray• Phosphoproteome (LC-MS/MS)	• Head and neck squamous carcinoma	• Intrinsic resistance• Identification of therapeutic targets	• Biological characterization	• Cisplatin	Niehr et al. (2018)
Network-based modeling					
• GCNA using mRNA microarray data	• Breast cancer	• Not specified	• Identification of biomarkers	• Trastuzumab and docetaxel	Li et al. (2021a)
• Cox regression model					
• Weighted GCNA using RNA-seq data	• Breast cancer	• Not specified	• Identification of biomarkers	• Doxorubicin	Li et al. (2021b)
• Cox regression model					
• Gene co-expression network analysis (GCNA) using RNA-seq data	• Breast cancer	• Not specified	• Identification of biomarkers	• Doxorubicin, cytoxan, and tamoxifen	Cui et al. (2020)
• Methylome (BeadChip array)					
• Genome sequencing			• Biological characterization		
• Weighted GCNA using mRNA microarray data	• Gastric cancer	• Acquired resistance	• Identification of biomarkers	• 5-FU and cisplatin	Qi and Zhang, (2020)
• ceRNA network for correlation between lncRNA and mRNA levels using RNA-seq data	• 19 Types (e.g., Lung cancer, breast cancer, and melanoma)	• Not specified	• Identification of biomarkers	• 138 Drugs (e.g., vorinostat and bosutinib)	Liu et al. (2019a)
			• Biological characterization		
• GCNA using RNA-seq data	• Glioma	• Acquired resistance	• Identification of biomarkers	• Dibutyryl cyclic adenosine monophosphate	Zhang et al. (2019)
• Cox regression model					
• Weighted GCNA using RNA-seq data	• Breast cancer	• Acquired resistance	• Identification of biomarkers	• Docetaxel	Huang et al. (2018)
Machine learning					
• Deep neural network (DNN) with neighborhood component analysis using CNV, somatic mutation, methylome, mRNA microarray, RNA-seq, and proteome data	• Breast cancer	• Not specified	• Prediction of a drug response	• 100 Drugs (e.g., tamoxifen)	Malik et al. (2021)
• Logistic regression using CNV, somatic mutation, mRNA microarray, drug targets, and drug descriptor data	• 955 Cell lines from GDSC (lung cancer, urogenital, and leukemia)	• Not specified	• Prediction of a drug response	• 219 Drugs (e.g., AT-7519) for GDSC cell lines	Yu et al. (2021)
	• 491 Cell lines from CCLE				
				• 24 Drugs (e.g., AZD6244) for CCLE cell lines	
• DNN with multiple elastic nets using mRNA microarray and drug descriptor data	• 983 Cell lines from GDSC	• Not specified	• Identification of biomarkers	• 222 Drugs (e.g., 5-FU) for GDSC cell lines	Choi et al. (2020)
	• 491 Cell lines from CCLE		• Prediction of a drug response	• 12 Drugs for CCLE cell lines	
• Weighted GCNA, elastic net, and random forest using proteome and phosphoproteome data	• NCI60 cell line panel	• Not specified	• Identification of biomarkers	• Various drugs (e.g., cytarabine, 5-FU)	Frejno et al. (2020)
• Cox regression model	• Prediction of a drug response				
			• CRC65 cell line panel		
• Ridge regression and support vector regression using mRNA microarray and RNA-seq data	• Colorectal cancer	• Not specified	• Identification of biomarkers	• 5-FU for colorectal cancer	Kong et al. (2020)
				• Cisplatin for bladder cancer	
	• Bladder cancer		• Prediction of a drug response		
• Ensemble transfer learning (LighGBM, or DNNs with two different architectures) using RNA-seq data and drug descriptor data	• Hundreds of cancer cell lines from CCLE, CTRP, gCSI and GDSC	• Not specified	• Prediction of a drug response	• Hundreds of drugs from CCLE, CTRP, gCSI and GDSC	Zhu et al. (2020)
• Artificial neural network using single-cell metabolome data	• Leukemia	• Intrinsic resistance	• Prediction of a drug response	• Cell adhesion as a indication of drug resistance without addition of a drug	Liu et al. (2019b)
• DNN using mRNA microarray and RNA-seq data	• 1,001 Cell lines from 55 tissues (e.g., leukemia) in GDSC	• Not specified	• Prediction of a drug response	• Bortezomib, PARP inhibitor, cisplatin, and paclitaxel	Sakellaropoulos et al. (2019)
• Random forest using RNA-seq, CNV, and methylome data	• Bladder cancer	• Not specified	• Identification of biomarkers	• Cisplatin and gemcitabine (for bladder cancer)	Xu et al. (2019)
			• Prediction of a drug response		
	• Glioma			• Temozolomide (for glioma)	
• Cox regression model					
	• Pancreatic cancer			• Gemcitabine (for pancreatic cancer)	
	• Gastric cancer			• 5-FU (for gastric cancer)	
• Elastic net using proteome and kinome data	• Colorectal cancer	• Not specified	• Identification of biomarkers	• 577 Drugs (e.g., cetuximab and afatinib)	Frejno et al. (2017)
• Cox regression model					
			• Prediction of a drug response		

## Multi-Omics Analyses

Multiple omics data are often generated to examine various biological aspects of drug-resistant cancer cells (Figure 1). Target genotypes and phenotypes examined using omics data (Table 1) include: cancer-associated mutations (Niehr et al., 2018; Marczyk et al., 2020; Sinkala et al., 2021); changes in the expression level of specific genes (Niehr et al., 2018; Nava et al., 2019; Kagohara et al., 2020; Marczyk et al., 2020; Poojan et al., 2020; Sinkala et al., 2021); changes in chromosome structure (Kagohara et al., 2020; Marczyk et al., 2020; Aissa et al., 2021); epigenetic alterations (e.g., methylation or acetylation states of histone proteins) (Nava et al., 2019; Kagohara et al., 2020; Marczyk et al., 2020; Poojan et al., 2020; Sinkala et al., 2021); and the presence of heterogeneity of a cell population (Niehr et al., 2018), often increasingly examined at a single-cell resolution (Kagohara et al., 2020; Aissa et al., 2021). In a recent study for cell line heterogeneity, for example, application of single-cell DNA and RNA sequencing (RNA-seq) to 20 triple-negative breast cancer (TNBC) patients revealed that rare pre-existing clones having genotypes associated with chemoresistance were adaptively selected in response to neoadjuvant chemotherapy, which subsequently led to acquired transcriptional reprogramming (Kim et al., 2018). For epigenetic alteration, chromosome conformation capture (Hi-C) along with additional omics analyses were conducted for estrogen receptor positive (ER+) breast cancer, which showed that resistance development to endocrine therapy was accompanied with notable 3-dimensional (3D) epigenome alterations (Achinger-Kawecka et al., 2020). Application of multi-omics analyses has also been extended to examine biological processes in quiescent cancer cells that show drug resistance (Lee et al., 2020; Kumar et al., 2021).

Understanding the biology of drug resistance often helps devise effective treatment strategies for drug-resistant cancer cells. Relevant examples (Table 1) include targeting: cancer stem cell phenotypes, in particular stem cell factor receptor c-KIT, for TNBC cells resistant to an anticancer agent RH1 that is currently under clinical trials (Kuciauskas et al., 2019); a range of biological pathways (e.g., metabolism), microenvironment as well as proliferation, migration and invasion of cells, which are all associated with drug resistance for diffuse large B-cell lymphoma patients (Fornecker et al., 2019); zinc finger MYND domain-containing protein 8 (ZMYND8), a putative chromatin reader that appeared to suppress tumorigenic potential and drug resistance induced by doxorubicin (Mukherjee et al., 2020); and EZH2 responsible for histone methylation in taxane-resistant TNBC (Deblois et al., 2020).

As representative examples of overcoming drug resistance on the basis of omics analyses, recent studies additionally conducted CRISPR-Cas9-based genetic screens to examine cellular plasticity, which was suggested as a therapeutic target for drug-resistant cancer cells (Bell et al., 2019; Torre et al., 2021). Cellular plasticity describes non-genetic transformation of a cellular state into a drug-resistant state by reprogramming gene expression profiles. In a study by Torre et al., CRISPR-Cas9 genetic screens were implemented for melanoma cells to identify genes that affect cell fate decisions by altering cellular plasticity (Torre et al., 2021). In particular, modulating the cellular plasticity was demonstrated for vemurafenib inhibiting B-Raf, encoded by a proto-oncogene, in melanoma. Interestingly, inhibiting DOT1L, associated with the onset of melanoma, before the B-Raf inhibition showed more drug resistance than simultaneous inhibition of DOT1 and B-Raf using pinometostat and vemurafenib, respectively. Subsequent transcriptome analysis of knockout cell lines generated clues for non-genetic mechanisms of drug resistance. Another study by Bell et al. focused on acute myeloid leukemia patients that showed non-genetic drug resistance (Bell et al., 2019). Single-cell RNA-seq, followed by CRISPR-Cas9 screening, led to the identification of genes responsible for transcriptional plasticity that triggered epigenetic resistance. Among the genes identified was Lsd1, the inhibition of which was shown to overcome non-genetic drug resistance. As demonstrated by these two recent studies, implementation of genome engineering in addition to omics analyses provides compelling evidence for targets that can help overcome drug resistance.

### COMPUTATIONAL MODELING APPROACHES

While various bioinformatic analyses are available for analyzing omics data, such as enrichment analyses, gene co-expression networks (GCNs) (Cui et al., 2020; Qi and Zhang, 2020) and their variants (e.g., a network of long non-coding RNAs and mRNAs) (Huang et al., 2018; Liu H. et al., 2019) as well as dimensionality reduction (e.g., t-SNE and UMAP), omics data have also been subjected to computational modeling to make predictions for discovering novel mechanisms and devising treatment strategies for drug-resistant cancers (Figure 1). Use of survival analysis in combination with GCNs, and development of a gene regulatory network (GRN) model using a set of ordinary differential equations (ODEs), machine learning models, and genome-scale metabolic models (GEMs) are representative computational modeling approaches that have recently been considered for studying drug-resistant cancer cells (Table 1).

### Network-Based Modeling

GCN has been a popular analysis for understanding gene expression patterns from transcriptome data. GCN is an undirected graph that can be constructed from transcriptome data (e.g., RNA-seq), and connects pairs of genes (nodes in a GCN) with an edge if each pair of genes shows significant co-expression patterns across the transcriptome data. GCN analysis, such as identifying hub genes and/or modules, allows prioritizing candidate genes that may be highly associated with drug resistance of cancer cells. Weighted GCN additionally considers the level of significance in the co-expression relationship between genes in a pair. Often, outcomes from (weighted) GCN analysis are further subjected to other computational analyses, for example survival analysis, to validate the biological and/or clinical significance of the candidate genes. As a recent example, Li et al. focused on *PPP2R2B*, encoding serine/threonine-protein phosphatase 2A 55 kDa regulatory subunit B beta isoform, as a potential prognostic biomarker for TNBC on the basis of a series of bioinformatic analyses involving a GCN (Li Z. et al., 2021). Kaplan-Meier survival analysis for this gene revealed that patients with a low expression level of *PPP2R2B* showed shorter survival time than those with a high expression level of *PPP2R2B*. Interestingly, *PPP2R2B* upregulation could attenuate the resistance of TNBC cells to doxorubicin. Likewise, Cox proportional hazards regression model (Cox regression model) was used for genes selected from GCNs to predict prognostic biomarkers for breast cancer, and to suggest genes (e.g., *CCNE2* and *KIF14*) that may help overcome drug resistance (Li Y.-K. et al., 2021).

While GCNs can provide clinically important information when combined with additional predictive models, such as survival analysis above, they have limitations in generating clues on a molecular mechanism associated with development of drug resistance, in particular dynamic interactions between genes. To address this problem, Zhang et al. developed a time-course RNA-seq data-driven computational framework (DryNetMC) to construct GRNs that help elucidate dynamic interactions between genes, and identify key genes associated with mechanisms of drug resistance (Zhang et al., 2019). DryNetMC involves a set of ODEs, a regularized regression method as well as a series of network analyses. Using DryNetMC, GRNs were constructed for dbcAMP-sensitive and dbcAMP-resistant glioma cells based on their time-course RNA-seq data. These differential GRNs were subsequently subjected to a systematic characterization to identify their unique network properties (e.g., node importance) that helped identify key genes (e.g., *KIF2C*, *CCNA2*, *NDC80*, *KIF11*, and *KIF23*) that are predictive of a cancer cell’s drug response. Because network-based models, either by using a GCN or other methods (e.g., ODEs), can visualize a biological context (e.g., association between genes), they will continue to be actively used in the analysis of omics data, and likely along with additional predictive models.

### Machine Learning

Increasing availability of omics data for drug-resistant cancer cells has also provided unprecedented opportunities for building machine learning models. In general, machine learning models perform classification or regression, depending on a given problem. Recently, prediction of anticancer drug response was attempted by using various types of machine learning methods, such as logistic regression (Frejno et al., 2017; Yu et al., 2021), random forest (Xu et al., 2019) and deep neural network (DNN; e.g., multilayer perceptron) (Malik et al., 2021) on the basis of a range of omics and drug response data (Table 1). When developing these machine learning models, transcriptome (RNA-seq or mRNA microarray) was the most frequently adopted dataset, but other types of datasets were also considered, including genome (e.g., gene mutations) (Yu et al., 2021), proteome (Frejno et al., 2020), epigenome (Xu et al., 2019), mass spectrometry data (Liu R. et al., 2019) and molecular features of a target drug (Zhu et al., 2020).

In a recent study by Kong et al., a machine learning model was developed that can predict a patient’s drug response on the basis of the analysis of protein-protein interaction (PPI) network and pharmacogenomic data from 3D organoid culture models (Kong et al., 2020). Specifically, potential biomarkers were first inferred from the PPI network analysis, and their corresponding expression profiles along with drug response data (IC_50_) were used to train a machine learning model (e.g., ridge regression). The resulting drug responses were validated using survival analysis by focusing on colorectal and bladder cancer patients treated with 5-fluorouracil and cisplatin, respectively. The predicted drug responses also appeared to be consistent with transcriptome profiles from drug-sensitive and drug-resistant isogenic cancer cell lines as well as data on somatic mutations associated with already known biomarkers. In this study, consideration of the network analysis not only helped improve the performance of the developed machine learning model, but also facilitated the interpretation of model prediction outcomes. Likewise, in another study, elastic net and random forest regression were used to predict drug responses from abundance data of proteins and their phosphorylation sites in cancer cell lines (Frejno et al., 2020).

Among machine learning methods, DNNs are increasingly used for various predictions, and they have also been used to predict drug responses. Sakellaropoulos et al. developed a DNN model by using GDSC datasets (i.e., transcriptomic data for 1,001 cancer cell lines and IC_50_ values of 251 drugs) to predict drug responses (Sakellaropoulos et al., 2019). Across several datasets tested, the DNN model showed consistently better performance than elastic net and random forest models. The DNN model was validated by conducting survival analyses for the model-predicted IC_50_ values, which split patients based on their drug responsiveness. Importantly, pathway enrichment analysis using information from the DNN model (i.e., weights that connect the input layer and the first hidden layer) appeared to associate specific biological pathways with mechanisms of action for drugs. In a more recent study, predicting drug response was also attempted by using a DNN model combined with multiple elastic nets (Choi et al., 2020), referred to as Reference Drug-based Neural Network (RefDNN). RefDNN was developed more in the context of drug resistance, which predicts whether a given cell line is resistant to a target drug by processing gene expression profiles and molecular structure of a drug. RefDNN was also shown to help identify biomarker genes associated with drug resistance, and explore a novel anticancer drug via drug repositioning.

Despite its demonstrated performance, machine learning is often challenged with the limited availability of training datasets for many technical fields. This challenge can be addressed to a certain extent by employing transfer learning as recently demonstrated (Zhu et al., 2020). Zhu et al. demonstrated that ensemble transfer learning can improve the prediction of drug responses in the context of drug repositioning (i.e., use of a drug for another cancer that is already known), precision oncology (i.e., use of a drug for a new cancer that has never been treated before) and new drug development (i.e., use of a new drug for already known cancer). In this particular study, LightGBM (Light Gradient Boosting Machine) and two different DNN models were considered for ensemble transfer learning; larger datasets from the CTRP and GDSC were used as source data for initial training of models, and smaller datasets from CCLE and the Genentech Cell Line Screening Initiative (gCSI) served as target data for further refinement and testing of the models. It was shown that ensemble transfer learning-based models almost always outperformed models that were not developed using transfer learning. This study suggests the use of transfer learning for other drug-resistant cancer cells where a training dataset is sufficiently not available.

### Genome-Scale Metabolic Modeling

GEM is a computational model that describes gene-protein-reaction (GPR) associations, and can be simulated to predict genome-scale metabolic flux distributions (Gu et al., 2019). GEMs are now available for an increasing number of organisms that are important in biotechnology and biomedicine. Several versions of human GEMs (Ryu et al., 2017; Brunk et al., 2018; Robinson et al., 2020) are currently available, which have been used to examine a target cell’s metabolism, and to predict biomarkers and drug targets for various diseases (Cook and Nielsen, 2017; Gu et al., 2019). For a medical application, a generic human GEM, covering all the known GPR associations in human metabolism, is initially integrated with omics data, often transcriptome (e.g., RNA-seq), to build a context-specific GEM, a GEM that is specific to a target cell or tissue (Ryu et al., 2015; Opdam et al., 2017). The resulting context-specific GEM is then simulated for various metabolic studies.

Human GEMs have recently been used to study radiation-resistant tumors (Lewis et al., 2021; Lewis and Kemp, 2021), but not drug-resistant cancer cells, to the best of our knowledge. Lewis et al. newly constructed GEMs for radiation-sensitive and radiation-resistant tumors through multi-omics integration (i.e., transcriptome data, mutational data, kinetic data and thermodynamic data) (Lewis et al., 2021). These context-specific GEMs were used to identify changes in redox cofactor production that give resistance to radiation therapy. In the other study, ensemble machine learning classifiers were developed to predict whether an individual is responsive or resistant to a radiation therapy by considering data of metabolite production rates predicted from context-specific GEMs as well as mutation data, transcriptome data and clinical data from TCGA (Lewis and Kemp, 2021). These two studies obviously suggest that GEM-based approaches can also be considered to identify metabolic signatures of drug-resistant cancer cells, and to predict effective drug targets for these cancer cells.

## Outlook

Understanding genotype-phenotype associations in drug-resistant cancer cells is a highly complex problem, and therefore use of multi-omics data has been considered to capture various aspects of these troubling cancer cells. In particular, multi-omics analyses along with additional tools, such as genome engineering (e.g., CRISPR-Cas9), will continue to play an important role in thorough characterization of drug-resistant cancer cells. Also, an increasing volume of omics data will facilitate development of various types of computational models. As a consequence, prediction outcomes from computational models will allow more systematically designing experiments for drug-resistant cancer cells.

Despite the promises of omics data and computational models, technical challenges exist. First, current coverage of multi-omics data is not sufficient for thoroughly studying a range of drug-resistant cancer cells. In particular, generation of a consistent set of multi-omics data from each single cell is necessary for in-depth study of a target cancer cell and comparison of different types of cancer cells. Also, it will be interesting to examine the effects of using datasets obtained from patients having a specific disease instead of publicly available datasets (e.g., GDSC and CTRP). While currently available machine learning models have been rigorously validated by using public datasets, they might reveal previously unnoticed limitations in a clinical setting because the public datasets are often generated in a highly controlled condition. In particular, additional consideration of non-genetic factors (e.g., age, gender, and lifestyle) may help reveal new insights on drug-resistant cancer cells. Use of patient-specific datasets will allow more widespread use of the state-of-the-art computational models in a clinical setting.

For network-based modeling, including both GCN and GRN, a breakthrough is needed that allows efficiently developing a cell-specific large-scale GRN that can be simulated under various conditions (e.g., gene perturbation). For machine learning, despite its high predictive performance, there is always a challenge of avoiding overfitting and achieving explainability. Explainability in terms of biological processes is particularly important in the field of biomedicine in order to explain prediction outcomes and make medical decisions. In case of human GEMs, because patient-specific omics data (e.g., RNA-seq) are available to a certain extent, human GEMs should be more actively considered to systematically examine metabolism of drug-resistant cancer cells. Availability of multi-omics data will be particularly useful for interpreting human GEMs and their prediction outcomes; because human GEMs only cover a metabolic network, use of multi-omics data can help explain a complex interplay between metabolic and regulatory networks. Prediction outcomes from the simulation of human GEMs will in turn help explain the insights reaped from omics analyses.

Taken together, advances in omics technologies and computational modeling will bring about positive impacts in understanding and treating drug-resistant cancer cells. Feedback from clinicians and biomedical researchers will be additionally useful for the successful development and clinical application of computational models.

## References

[B1] Achinger-Kawecka J. Valdes-Mora F. Luu P.-L. Giles K. A. Caldon C. E. Qu W. (2020). Epigenetic Reprogramming at Estrogen-Receptor Binding Sites Alters 3D Chromatin Landscape in Endocrine-Resistant Breast Cancer. Nat. Commun. 11 (1), 320. 10.1038/s41467-019-14098-x 31949157 PMC6965612

[B2] Aissa A. F. Islam A. B. M. M. K. Ariss M. M. Go C. C. Rader A. E. Conrardy R. D. (2021). Single-cell Transcriptional Changes Associated with Drug Tolerance and Response to Combination Therapies in Cancer. Nat. Commun. 12 (1), 1628. 10.1038/s41467-021-21884-z 33712615 PMC7955121

[B3] Bell C. C. Fennell K. A. Chan Y.-C. Rambow F. Yeung M. M. Vassiliadis D. (2019). Targeting Enhancer Switching Overcomes Non-genetic Drug Resistance in Acute Myeloid Leukaemia. Nat. Commun. 10 (1), 2723. 10.1038/s41467-019-10652-9 31222014 PMC6586637

[B4] Brunk E. Sahoo S. Zielinski D. C. Altunkaya A. Dräger A. Mih N. (2018). Recon3D Enables a Three-Dimensional View of Gene Variation in Human Metabolism. Nat. Biotechnol. 36 (3), 272–281. 10.1038/nbt.4072 29457794 PMC5840010

[B5] Bukowski K. Kciuk M. Kontek R. (2020). Mechanisms of Multidrug Resistance in Cancer Chemotherapy. Ijms 21 (9), 3233. 10.3390/ijms21093233 PMC7247559 32370233

[B6] Cai Q. Wang S. Jin L. Weng M. Zhou D. Wang J. (2019). Long Non-coding RNA GBCDRlnc1 Induces Chemoresistance of Gallbladder Cancer Cells by Activating Autophagy. Mol. Cancer 18 (1), 82. 10.1186/s12943-019-1016-0 30953511 PMC6449938

[B7] Choi J. Park S. Ahn J. (2020). RefDNN: a Reference Drug Based Neural Network for More Accurate Prediction of Anticancer Drug Resistance. Sci. Rep. 10 (1), 1861. 10.1038/s41598-020-58821-x 32024872 PMC7002431

[B8] Collisson E. A. Weinstein J. N. Weinstein J. N. Mills G. B. Shaw K. R. M. Ozenberger B. A. (2013). The Cancer Genome Atlas Pan-Cancer Analysis Project. Nat. Genet. 45 (10), 1113–1120. 10.1038/ng.2764 24071849 PMC3919969

[B9] Consortium I. T. P.-C. A. o. W. G. (2020). Pan-cancer Analysis of Whole Genomes. Nature 578 (7793), 82–93. 10.1038/s41586-020-1969-6 32025007 PMC7025898

[B10] Cook D. J. Nielsen J. (2017). Genome-scale Metabolic Models Applied to Human Health and Disease. Wires Syst. Biol. Med. 9 (6), e1393. 10.1002/wsbm.1393 28644920

[B11] Costa D. B. Nguyen K.-S. H. Cho B. C. Sequist L. V. Jackman D. M. Riely G. J. (2008). Effects of Erlotinib in EGFR Mutated Non-small Cell Lung Cancers with Resistance to Gefitinib. Clin. Cancer Res. 14 (21), 7060–7067. 10.1158/1078-0432.CCR-08-1455 18981003 PMC2596582

[B12] Cui H. Kong H. Peng F. Wang C. Zhang D. Tian J. (2020). Inferences of Individual Drug Response-Related Long Non-coding RNAs Based on Integrating Multi-Omics Data in Breast Cancer. Mol. Ther. - Nucleic Acids 20, 128–139. 10.1016/j.omtn.2020.01.038 32163894 PMC7066040

[B13] De Luca A. Parker L. J. Ang W. H. Rodolfo C. Gabbarini V. Hancock N. C. (2019). A Structure-Based Mechanism of Cisplatin Resistance Mediated by Glutathione Transferase P1-1. Proc. Natl. Acad. Sci. USA 116 (28), 13943–13951. 10.1073/pnas.1903297116 31221747 PMC6628828

[B14] Deblois G. Tonekaboni S. A. M. Grillo G. Martinez C. Kao Y. I. Tai F. (2020). Epigenetic Switch-Induced Viral Mimicry Evasion in Chemotherapy-Resistant Breast Cancer. Cancer Discov. 10 (9), 1312–1329. 10.1158/2159-8290.CD-19-1493 32546577

[B15] Fischer K. R. Durrans A. Lee S. Sheng J. Li F. Wong S. T. C. (2015). Epithelial-to-mesenchymal Transition Is Not Required for Lung Metastasis but Contributes to Chemoresistance. Nature 527 (7579), 472–476. 10.1038/nature15748 26560033 PMC4662610

[B16] Fornecker L.-M. Muller L. Bertrand F. Paul N. Pichot A. Herbrecht R. (2019). Multi-omics Dataset to Decipher the Complexity of Drug Resistance in Diffuse Large B-Cell Lymphoma. Sci. Rep. 9 (1), 895. 10.1038/s41598-018-37273-4 30696890 PMC6351558

[B17] Frejno M. Meng C. Ruprecht B. Oellerich T. Scheich S. Kleigrewe K. (2020). Proteome Activity Landscapes of Tumor Cell Lines Determine Drug Responses. Nat. Commun. 11 (1), 3639. 10.1038/s41467-020-17336-9 32686665 PMC7371697

[B18] Frejno M. Zenezini Chiozzi R. Wilhelm M. Koch H. Zheng R. Klaeger S. (2017). Pharmacoproteomic Characterisation of Human colon and Rectal Cancer. Mol. Syst. Biol. 13 (11), 951. 10.15252/msb.20177701 29101300 PMC5731344

[B19] Gatti L. Zunino F. (2005). Overview of Tumor Cell Chemoresistance Mechanisms. Methods Mol. Med. 111, 127–148. 10.1385/1-59259-889-7:127 15911977

[B20] Ghandi M. Huang F. W. Jané-Valbuena J. Kryukov G. V. Lo C. C. Barretina J. (2019). Next-generation Characterization of the Cancer Cell Line Encyclopedia. Nature 569 (7757), 503–508. 10.1038/s41586-019-1186-3 31068700 PMC6697103

[B21] Giddings E. L. Champagne D. P. Wu M.-H. Laffin J. M. Thornton T. M. Valenca-Pereira F. (2021). Mitochondrial ATP Fuels ABC Transporter-Mediated Drug Efflux in Cancer Chemoresistance. Nat. Commun. 12 (1), 2804. 10.1038/s41467-021-23071-6 33990571 PMC8121950

[B22] Gu C. Kim G. B. Kim W. J. Kim H. U. Lee S. Y. (2019). Current Status and Applications of Genome-Scale Metabolic Models. Genome Biol. 20 (1), 121. 10.1186/s13059-019-1730-3 31196170 PMC6567666

[B23] Harte M. T. Gorski J. J. Savage K. I. Purcell J. W. Barros E. M. Burn P. M. (2014). NF-κB Is a Critical Mediator of BRCA1-Induced Chemoresistance. Oncogene 33 (6), 713–723. 10.1038/onc.2013.10 23435429 PMC3917825

[B24] Housman G. Byler S. Heerboth S. Lapinska K. Longacre M. Snyder N. (2014). Drug Resistance in Cancer: an Overview. Cancers 6 (3), 1769–1792. 10.3390/cancers6031769 25198391 PMC4190567

[B25] Huang P. Li F. Li L. You Y. Luo S. Dong Z. (2018). lncRNA Profile Study Reveals the mRNAs and lncRNAs Associated with Docetaxel Resistance in Breast Cancer Cells. Sci. Rep. 8 (1), 17970. 10.1038/s41598-018-36231-4 30568280 PMC6299474

[B26] Jiang Y. Cheng J. Yang C. Hu Y. Li J. Han Y. (2017). An Ultrasensitive Fluorogenic Probe for Revealing the Role of Glutathione in Chemotherapy Resistance. Chem. Sci. 8 (12), 8012–8018. 10.1039/c7sc03338a 29568448 PMC5853925

[B27] Kagohara L. T. Zamuner F. Davis-Marcisak E. F. Sharma G. Considine M. Allen J. (2020). Integrated Single-Cell and Bulk Gene Expression and ATAC-Seq Reveals Heterogeneity and Early Changes in Pathways Associated with Resistance to Cetuximab in HNSCC-Sensitive Cell Lines. Br. J. Cancer 123 (1), 101–113. 10.1038/s41416-020-0851-5 32362655 PMC7341752

[B28] Kim C. Gao R. Sei E. Brandt R. Hartman J. Hatschek T. (2018). Chemoresistance Evolution in Triple-Negative Breast Cancer Delineated by Single-Cell Sequencing. Cell 173 (4), 879–893. e813 10.1016/j.cell.2018.03.041 29681456 PMC6132060

[B29] Kong J. Lee H. Kim D. Han S. K. Ha D. Shin K. (2020). Network-based Machine Learning in Colorectal and Bladder Organoid Models Predicts Anti-cancer Drug Efficacy in Patients. Nat. Commun. 11 (1), 5485. 10.1038/s41467-020-19313-8 33127883 PMC7599252

[B30] Kuciauskas D. Dreize N. Ger M. Kaupinis A. Zemaitis K. Stankevicius V. (2019). Proteomic Analysis of Breast Cancer Resistance to the Anticancer Drug RH1 Reveals the Importance of Cancer Stem Cells. Cancers 11 (7), 972. 10.3390/cancers11070972 PMC6678540 31336714

[B31] Kumar S. Nandi A. Singh S. Regulapati R. Li N. Tobias J. W. (2021). Dll1+ Quiescent Tumor Stem Cells Drive Chemoresistance in Breast Cancer through NF-Κb Survival Pathway. Nat. Commun. 12 (1), 432. 10.1038/s41467-020-20664-5 33462238 PMC7813834

[B32] Lee S. Micalizzi D. Truesdell S. S. Bukhari S. I. A. Boukhali M. Lombardi-Story J. (2020). A post-transcriptional Program of Chemoresistance by AU-Rich Elements and TTP in Quiescent Leukemic Cells. Genome Biol. 21 (1), 33. 10.1186/s13059-020-1936-4 32039742 PMC7011231

[B33] Lewis J. E. Forshaw T. E. Boothman D. A. Furdui C. M. Kemp M. L. (2021). Personalized Genome-Scale Metabolic Models Identify Targets of Redox Metabolism in Radiation-Resistant Tumors. Cel Syst. 12 (1), 68–81. e11 10.1016/j.cels.2020.12.001 PMC7905848 33476554

[B34] Lewis J. E. Kemp M. L. (2021). Integration of Machine Learning and Genome-Scale Metabolic Modeling Identifies Multi-Omics Biomarkers for Radiation Resistance. Nat. Commun. 12 (1), 2700. 10.1038/s41467-021-22989-1 33976213 PMC8113601

[B35] Li Y.-K. Hsu H.-M. Lin M.-C. Chang C.-W. Chu C.-M. Chang Y.-J. (2021a). Genetic Co-expression Networks Contribute to Creating Predictive Model and Exploring Novel Biomarkers for the Prognosis of Breast Cancer. Sci. Rep. 11 (1), 7268. 10.1038/s41598-021-84995-z 33790307 PMC8012617

[B36] Li Z. Li Y. Wang X. Yang Q. (2021b). PPP2R2B Downregulation Is Associated with Immune Evasion and Predicts Poor Clinical Outcomes in Triple-Negative Breast Cancer. Cancer Cel Int 21 (1), 13. 10.1186/s12935-020-01707-9 PMC7788839 33407498

[B37] Likhite V. S. Stossi F. Kim K. Katzenellenbogen B. S. Katzenellenbogen J. A. (2006). Kinase-specific Phosphorylation of the Estrogen Receptor Changes Receptor Interactions with Ligand, Deoxyribonucleic Acid, and Coregulators Associated with Alterations in Estrogen and Tamoxifen Activity. Mol. Endocrinol. 20 (12), 3120–3132. 10.1210/me.2006-0068 16945990

[B38] Lim Z.-F. Ma P. C. (2019). Emerging Insights of Tumor Heterogeneity and Drug Resistance Mechanisms in Lung Cancer Targeted Therapy. J. Hematol. Oncol. 12 (1), 134. 10.1186/s13045-019-0818-2 31815659 PMC6902404

[B39] Liu H. Wang S. Zhou S. Meng Q. Ma X. Song X. (2019a). Drug Resistance-Related Competing Interactions of lncRNA and mRNA across 19 Cancer Types. Mol. Ther. - Nucleic Acids 16, 442–451. 10.1016/j.omtn.2019.03.011 31048183 PMC6488743

[B40] Liu R. Zhang G. Yang Z. (2019b). Towards Rapid Prediction of Drug-Resistant Cancer Cell Phenotypes: Single Cell Mass Spectrometry Combined with Machine Learning. Chem. Commun. 55 (5), 616–619. 10.1039/c8cc08296k PMC6640148 30525135

[B41] Malik V. Kalakoti Y. Sundar D. (2021). Deep Learning Assisted Multi-Omics Integration for Survival and Drug-Response Prediction in Breast Cancer. BMC Genomics 22 (1), 214. 10.1186/s12864-021-07524-2 33761889 PMC7992339

[B42] Marczyk M. Patwardhan G. A. Zhao J. Qu R. Li X. Wali V. B. (2020). Multi-Omics Investigation of Innate Navitoclax Resistance in Triple-Negative Breast Cancer Cells. Cancers 12 (9), 2551. 10.3390/cancers12092551 PMC7563413 32911681

[B43] Marine J.-C. Dawson S.-J. Dawson M. A. (2020). Non-genetic Mechanisms of Therapeutic Resistance in Cancer. Nat. Rev. Cancer 20 (12), 743–756. 10.1038/s41568-020-00302-4 33033407

[B44] Martin S. Dudek-Peric A. M. Garg A. D. Roose H. Demirsoy S. Van Eygen S. (2017). An Autophagy-Driven Pathway of ATP Secretion Supports the Aggressive Phenotype of BRAFV600E Inhibitor-Resistant Metastatic Melanoma Cells. Autophagy 13 (9), 1512–1527. 10.1080/15548627.2017.1332550 28722539 PMC5612289

[B45] Mukherjee S. Adhikary S. Gadad S. S. Mondal P. Sen S. Choudhari R. (2020). Suppression of Poised Oncogenes by ZMYND8 Promotes Chemo-Sensitization. Cell Death Dis 11 (12), 1073. 10.1038/s41419-020-03129-x 33323928 PMC7738522

[B46] Nava M. Dutta P. Farias-Eisner R. Vadgama J. V. Wu Y. (2019). Utilization of NGS Technologies to Investigate Transcriptomic and Epigenomic Mechanisms in Trastuzumab Resistance. Sci. Rep. 9 (1), 5141. 10.1038/s41598-019-41672-6 30914750 PMC6435657

[B47] Niehr F. Eder T. Pilz T. Konschak R. Treue D. Klauschen F. (2018). Multilayered Omics-Based Analysis of a Head and Neck Cancer Model of Cisplatin Resistance Reveals Intratumoral Heterogeneity and Treatment-Induced Clonal Selection. Clin. Cancer Res. 24 (1), 158–168. 10.1158/1078-0432.CCR-17-2410 29061642

[B48] Opdam S. Richelle A. Kellman B. Li S. Zielinski D. C. Lewis N. E. (2017). A Systematic Evaluation of Methods for Tailoring Genome-Scale Metabolic Models. Cel Syst. 4 (3), 318–329. 10.1016/j.cels.2017.01.010 PMC5526624 28215528

[B49] Poojan S. Bae S.-H. Min J.-W. Lee E. Y. Song Y. Kim H. Y. (2020). Cancer Cells Undergoing Epigenetic Transition Show Short-Term Resistance and Are Transformed into Cells with Medium-Term Resistance by Drug Treatment. Exp. Mol. Med. 52 (7), 1102–1115. 10.1038/s12276-020-0464-3 32661348 PMC8080688

[B50] Qi W. Zhang Q. (2020). Gene's Co-expression Network and Experimental Validation of Molecular Markers Associated with the Drug Resistance of Gastric Cancer. Biomarkers Med. 14 (9), 761–773. 10.2217/bmm-2019-0504 32715733

[B51] Robinson J. L. Kocabaş P. Wang H. Cholley P.-E. Cook D. Nilsson A. (2020). An Atlas of Human Metabolism. Sci. Signal. 13 (624), eaaz1482. 10.1126/scisignal.aaz1482 32209698 PMC7331181

[B52] Ryu J. Y. Kim H. U. Lee S. Y. (2017). Framework and Resource for More Than 11,000 Gene-Transcript-Protein-Reaction Associations in Human Metabolism. Proc. Natl. Acad. Sci. USA 114 (45), E9740–E9749. 10.1073/pnas.1713050114 29078384 PMC5692585

[B53] Ryu J. Y. Kim H. U. Lee S. Y. (2015). Reconstruction of Genome-Scale Human Metabolic Models Using Omics Data. Integr. Biol. (Camb.) 7 (8), 859–868. 10.1039/c5ib00002e 25730289

[B54] Sakellaropoulos T. Vougas K. Narang S. Koinis F. Kotsinas A. Polyzos A. (2019). A Deep Learning Framework for Predicting Response to Therapy in Cancer. Cel Rep. 29 (11), 3367–3373. e3364 10.1016/j.celrep.2019.11.017 31825821

[B55] Seashore-Ludlow B. Rees M. G. Cheah J. H. Cokol M. Price E. V. Coletti M. E. (2015). Harnessing Connectivity in a Large-Scale Small-Molecule Sensitivity Dataset. Cancer Discov. 5 (11), 1210–1223. 10.1158/2159-8290.CD-15-0235 26482930 PMC4631646

[B56] Seth S. Li C.-Y. Ho I.-L. Corti D. Loponte S. Sapio L. (2019). Pre-existing Functional Heterogeneity of Tumorigenic Compartment as the Origin of Chemoresistance in Pancreatic Tumors. Cel Rep. 26 (6), 1518–1532. e1519 10.1016/j.celrep.2019.01.048 30726735

[B57] Shoemaker R. H. (2006). The NCI60 Human Tumour Cell Line Anticancer Drug Screen. Nat. Rev. Cancer 6 (10), 813–823. 10.1038/nrc1951 16990858

[B58] Sinkala M. Nkhoma P. Mulder N. Martin D. P. (2021). Integrated Molecular Characterisation of the MAPK Pathways in Human Cancers Reveals Pharmacologically Vulnerable Mutations and Gene Dependencies. Commun. Biol. 4 (1), 9. 10.1038/s42003-020-01552-6 33398072 PMC7782843

[B59] Subramanian A. Narayan R. Corsello S. M. Peck D. D. Natoli T. E. Lu X. (2017). A Next Generation Connectivity Map: L1000 Platform and the First 1,000,000 Profiles. Cell 171 (6), 1437–1452. e1417 10.1016/j.cell.2017.10.049 29195078 PMC5990023

[B60] Tate J. G. Bamford S. Jubb H. C. Sondka Z. Beare D. M. Bindal N. (2019). COSMIC: the Catalogue of Somatic Mutations in Cancer. Nucleic Acids Res. 47 (D1), D941–D947. 10.1093/nar/gky1015 30371878 PMC6323903

[B61] Torre E. A. Arai E. Bayatpour S. Jiang C. L. Beck L. E. Emert B. L. (2021). Genetic Screening for Single-Cell Variability Modulators Driving Therapy Resistance. Nat. Genet. 53 (1), 76–85. 10.1038/s41588-020-00749-z 33398196 PMC7796998

[B62] Vasan N. Baselga J. Hyman D. M. (2019). A View on Drug Resistance in Cancer. Nature 575 (7782), 299–309. 10.1038/s41586-019-1730-1 31723286 PMC8008476

[B63] Xu Y. Dong Q. Li F. Xu Y. Hu C. Wang J. (2019). Identifying Subpathway Signatures for Individualized Anticancer Drug Response by Integrating Multi-Omics Data. J. Transl. Med. 17 (1), 255. 10.1186/s12967-019-2010-4 31387579 PMC6685260

[B64] Yang W. Soares J. Greninger P. Edelman E. J. Lightfoot H. Forbes S. (2013). Genomics of Drug Sensitivity in Cancer (GDSC): a Resource for Therapeutic Biomarker Discovery in Cancer Cells. Nucleic Acids Res. 41 (Database issue), D955–D961. 10.1093/nar/gks1111 23180760 PMC3531057

[B65] Yu L. Zhou D. Gao L. Zha Y. (2021). Prediction of Drug Response in Multilayer Networks Based on Fusion of Multiomics Data. Methods 192, 85–92. 10.1016/j.ymeth.2020.08.006 32798653

[B66] Zhang J. Zhu W. Wang Q. Gu J. Huang L. F. Sun X. (2019). Differential Regulatory Network-Based Quantification and Prioritization of Key Genes Underlying Cancer Drug Resistance Based on Time-Course RNA-Seq Data. Plos Comput. Biol. 15 (11). e1007435 10.1371/journal.pcbi.1007435 31682596 PMC6827891

[B67] Zhao Y. Li Z. X. Zhu Y. J. Fu J. Zhao X. F. Zhang Y. N. (2021). Single‐Cell Transcriptome Analysis Uncovers Intratumoral Heterogeneity and Underlying Mechanisms for Drug Resistance in Hepatobiliary Tumor Organoids. Adv. Sci. 8 (11). 2003897 10.1002/advs.202003897 PMC8188185 34105295

[B68] Zheng H.-C. (2017). The Molecular Mechanisms of Chemoresistance in Cancers. Oncotarget 8 (35), 59950–59964. 10.18632/oncotarget.19048 28938696 PMC5601792

[B69] Zheng X. Carstens J. L. Kim J. Scheible M. Kaye J. Sugimoto H. (2015). Epithelial-to-mesenchymal Transition Is Dispensable for Metastasis but Induces Chemoresistance in Pancreatic Cancer. Nature 527 (7579), 525–530. 10.1038/nature16064 26560028 PMC4849281

[B70] Zhu Y. Brettin T. Evrard Y. A. Partin A. Xia F. Shukla M. (2020). Ensemble Transfer Learning for the Prediction of Anti-cancer Drug Response. Sci. Rep. 10 (1), 18040. 10.1038/s41598-020-74921-0 33093487 PMC7581765

